# Human L-ficolin, a Recognition Molecule of the Lectin Activation Pathway of Complement, Activates Complement by Binding to Pneumolysin, the Major Toxin of *Streptococcus pneumoniae*

**DOI:** 10.1371/journal.pone.0082583

**Published:** 2013-12-12

**Authors:** Youssif M. Ali, Hany I. Kenawy, Adnan Muhammad, Robert B. Sim, Peter W. Andrew, Wilhelm J. Schwaeble

**Affiliations:** 1 Department of Infection, Immunity and Inflammation, University of Leicester, Leicester, United Kingdom; 2 Department of Microbiology, Faculty of Pharmacy, Mansoura University, Mansoura, Egypt; University Medical Center Utrecht, The Netherlands

## Abstract

The complement system is an essential component of the immune response, providing a critical line of defense against different pathogens including *S. pneumoniae*. Complement is activated via three distinct pathways: the classical (CP), the alternative (AP) and the lectin pathway (LP). The role of Pneumolysin (PLY), a bacterial toxin released by *S. pneumoniae*, in triggering complement activation has been studied *in vitro*. Our results demonstrate that in both human and mouse sera complement was activated via the CP, initiated by direct binding of even non-specific IgM and IgG3 to PLY. Absence of CP activity in C1q^−/−^ mouse serum completely abolished any C3 deposition. However, C1q depleted human serum strongly opsonized PLY through abundant deposition of C3 activation products, indicating that the LP may have a vital role in activating the human complement system on PLY. We identified that human L-ficolin is the critical LP recognition molecule that drives LP activation on PLY, while all of the murine LP recognition components fail to bind and activate complement on PLY. This work elucidates the detailed interactions between PLY and complement and shows for the first time a specific role of the LP in PLY-mediated complement activation in human serum.

## Introduction

*Streptococcus pneumoniae* (the pneumococcus) is a Gram-positive bacterium that causes a wide range of diseases in humans [1]. Despite advances in antibiotic therapy and vaccination, pneumococcal diseases still represent a major public health challenge [2]. Pneumococcal diseases result in substantial morbidity and mortality, especially in young children and patients over 65 years of age [3]. In order to establish infection, *S. pneumoniae* has to have effective mechanisms to escape the host immune defense, especially at the first line of the humoral immune response provided by the complement system [2].

Complement activation is mediated via three distinct pathways; the classical (CP), the alternative (AP) and the lectin pathway (LP). The CP is initiated by the binding of C1q to charge clusters on targets or to antigen-antibody complexes. Thus, activation may depend either on the presence of natural antibodies in the non-immune host (primarily IgM or IgG with relatively low affinity to bind to specific antigens) or affinity-matured, specific immunoglobulins of the immunoglobulin classes IgM or IgG1, IgG2 or IgG3 in immune sera. LP activation is initiated via several LP specific carbohydrate recognition subcomponents, such as mannan binding lectin (MBL), ficolins and collectin 11 (CL-11) [4]. The alternative pathway can be activated by both antibody-dependent and independent routes, and acts as an amplification loop for both the classical and the lectin pathways [5]. The LP recognition molecules, MBL and CL-11, recognize carbohydrate residues on the surface of different microorganisms, while ficolins primarily recognize and drive LP activation on acetylated ligand structures. This diversity in the recognition of different carbohydrate residues broadens the range of microbial structures that initiate and activate the LP and provides protection against a wide range of microorganisms [4].

Initiation of complement activation leads to the formation of C3 convertases of either the CP or the LP (i.e. C4b2a) and/or the AP (i.e. C3bBb). These C3 convertases cleave the abundant plasma protein C3 into C3a and C3b. While C3a serves as an anaphylatoxin, C3b and its factor I-processed cleavage product iC3b are bound to the surface of microorganisms and serve as opsonins by facilitating the phagocytosis and killing of pathogens like *S. pneumoniae* through C3-receptor bearing phagocytes [4]. Complement C3b and iC3b deposition on *S. pneumoniae* can lead to the clearance of this pathogen through enhanced opsonophagocytosis which constitutes a key event in the innate and adaptive immune response against the pneumococcus [4]. Consequently, the pneumococcus has evolved several mechanisms to resist complement-mediated immunity. The capsular polysaccharides play a key role in the protection of pneumococci from complement attack and opsonophagocytosis because they may to some extent prevent complement C3 deposition and CP activation on the pathogen surface by masking or hiding bacterial surface antigens from natural IgM [6], [7]. In addition to the capsular polysaccharides, the pneumococcal surface proteins A and C (PspA, PspC) have been identified as being involved in the inhibition of complement deposition on the surface of *S. pneumoniae*. Both inhibit C4 deposition on the bacterial surface by sequestering the complement regulatory components factor H (FH) and C4-binding protein (C4bp) from host plasma. PspA and PspC serve as potent cofactors in the factor I-mediated conversion of C4b to iC4b (N.B. iC4b has lost the ability to form a C3 convertase and therefore C4b cleavage inhibits subsequent complement activation). This in turn impairs complement activation on the surface of *S. pneumoniae* by the CP [8], [9], while the LP can still opsonize the pneumococcal surface with C3 activation products (C3b and iC3b) since MASP-2 can activate C3 in the absence of C4b through a C4-bypass activation route [4]. The pneumococcal toxin PLY is also considered to contribute towards pathogen evasion from the complement-mediated clearance by directing complement activation away from bacterial surface. Mutant pneumococcal strains deficient in PLY showed increased C3 deposition on the bacterial cell surface and reduced pathogenicity, indicating an important role of PLY in the pathogenesis of *S. pneumoniae* infection [2].

PLY is a potent multifunctional virulence factor produced by all serotypes of *S. pneumoniae* as a 52 kDa soluble monomer that is capable of binding to cholesterol-rich membranes [10]. Upon binding, PLY monomers oligomerize to form a pore in the host cell membrane, which mediate cell lysis and cell death [11]. In addition, at sublytic concentrations, PLY was shown to have various effects on host cells: for example, PLY has been found to inhibit ciliary beating of the respiratory epithelium in mice and to inhibit phagocytosis and induce cytokine synthesis. PLY was also shown to mediate CD4^+^ T cell activation and chemotaxis [11], [12].

PLY was previously shown to activate complement via the CP independently of the presence of PLY-specific antibodies, a mechanism considered to play an important role in the pathogenesis of *S. pneumoniae* infection [2], [13], [24]. We show that C1q fails to bind to PLY directly and its binding requires the presence of immunoglobulines binding to PLY via their Fc regions. Our present analysis identified that either IgM or the IgG subclass IgG3 mediate this effect. PLY-bound IgM and IgG3 offer multiple binding interaction sites for the globular heads of C1q to initiate complement activation via subsequent activation of the C1q-associated serine protease tetramer C1s-C1r-C1r-C1s embedded within the dome structure formed by the hexameric C1q macromolecule [14], [15].

Furthermore, we show for the first time that PLY activates the LP of complement in human serum by direct binding interactions between PLY and the human LP recognition subcomponent L-ficolin, while the mouse L-ficolin orthologue ficolin-A (as well as any of the other murine LP recognition subcomponents) fails to bind and activate the LP on PLY.

## Materials and Methods

### Ethics statement

All animal procedures were authorized by the UK Home Office (Animals Scientific Procedures Act 1986; Home Office project license (60/4327)) and approved by the University of Leicester animal welfare committee. Every effort was made to minimize suffering of mice and mice were immediately culled by cervical dislocation after cardiac puncture. Human blood was obtained from healthy adult donors who provided written and informed consent, as required by the local ethics committee at the University of Leicester.

### Chemicals and Reagents

Unless otherwise stated, all chemicals and reagents were obtained from Sigma-Aldrich. PSA, a polysaccharide produced by *Aerococcus viridans* that binds to H-ficolin (alias FCN3), was prepared as previously described [16]. OMS721, a recombinant monoclonal antibody (mAb) that potently inhibits human MASP-2 functional activity, has been described previously and was kindly provided by OMEROS Corporation, Seattle, USA [17].

Anti ficolin-A antibodies were kindly provided by Professor T. Fujita, Fukushima University, Japan. Mouse anti-human CL-11, rat anti-mouse CL-11 mAb, rabbit anti-human C1q were kindly provided by Dr. Soren Hansen, Center for Medical Biotechnology Institute of Medical Biology, University of Southern Denmark, Odense, Denmark.

Blood was collected from MASP-2^−/−^, C1q^−/−^ or wild type mice. Mouse blood was taken by cardiac puncture under anesthesia. Mice were culled immediately by cervical dislocation after cardiac puncture. Blood was allowed to clot on ice for 2 hrs and serum was separated by centrifugation and kept as aliquots at −80°C. Human C1q depleted serum was purchased from Quidel, USA. Purified human and mouse C1q were prepared as previously described by [18].

### Cloning, expression, and purification of recombinant PLY

The open reading frame (ORF) of PLY was amplified from the genomic DNA of *S. pneumoniae* D39 using forward (5′ GGATCCACTCTTGACCCATCAGGGAGAAAGTATTG 3′) and reverse (5′ GGTACCCTAGTCATTTTCTACCTTATCCTC 3′) primers. The stop codon of the PLY ORF was mutated to be in frame with the 6-histidine tag provided by the bacterial expression vector pRsetB (Promega). Recombinant protein with 6-histidine tag was expressed and purified as previously described [19].

### Solid phase binding assays

Nunc Maxisorb microtiter plates were coated with 100 µl of the following reagents: 10 µg/ml mannan (a control for MBL binding), 10 µg/ml zymosan (control for CL-11 binding), 10 µg/ml N-acetylated BSA (Promega; a control for Ficolin-A and L-ficolin (FCN2) binding), and 10 µg/ml PSA (control for FCN3), 10 µg/ml PLY in coating buffer (15 mM Na_2_CO_3_, 35 mM NaHCO_3_, pH 9.6). For C1q binding assays, an *in situ* immune complex was prepared by coating plates with human serum albumin (HSA) then incubating with goat anti HSA (Scottish antibody production unit). Wells were blocked with 250 µl of 1% (w/v) BSA in TBS buffer (10 mM Tris-HCl, 140 mM NaCl, pH 7.4), and then washed three times with 250 µl of TBS with 0.05% (v/v) Tween 20 and 5 mM CaCl_2_ (wash buffer). Serial dilutions of serum in 100 µl of BBS (4 mM barbital, 145 mM NaCl, 2 mM CaCl_2_, 1 mM MgCl_2_, pH 7.4) were added and the plates were incubated for 1 hr at room temperature. Plates were washed and bound proteins were detected using monoclonal rat anti-mouse MBL-A (Hycult), rat anti-mouse MBL-C (Hycult), rabbit anti-mouse ficolin-A, rabbit anti-human L-ficolin (Sigma), mouse anti-human H-ficolin (Hycult), mouse anti-human CL-11, rat anti-mouse CL-11 mAb, rabbit anti-human C1q.

For detection of IgG and IgM binding to PLY, different IgG isotypes (IgG1, IgG2, IgG3 (Sigma)) and IgM were serially diluted in BBS buffer and incubated with PLY-coated plates. Secondary antibodies were alkaline phosphatase-conjugates (Sigma). Bound antibodies were detected using the chromogenic substrate p-nitrophenylphosphate (pNPP). The developed color was measured at wavelength 405 nm [4].

### Complement activation assay

Complement activation was measured by the detection of C3b deposition. To measure C3b deposition, Nunc MaxiSorb microtiter plates were coated with 100 µl of 10 µg/ml of mannan (Promega), or recombinant PLY in coating buffer. After overnight incubation, wells were blocked with 0.1% (w/v) HSA in TBS and then washed. Serum samples were diluted in BBS buffer, added to the plates and incubated for 1 hour at 37°C. Plates were washed and bound C3b was detected using rabbit anti-human C3c (Dako) followed by alkaline phosphatase-conjugated goat anti-rabbit IgG (Sigma). Bound antibody was detected using the chromogenic substrate pNPP [20].

### Fluid-phase PLY-induced complement C3 activation

Activation of C3 by PLY was assessed using Western blotting analysis as previously described [8]. Briefly, 1.25% (v/v) human serum was incubated with serial dilutions of PLY BBS buffer for 1 hr at 37°C starting with 100 µg/ml. Samples of this reaction were taken at different time points and analysed for C3 activation on 10% SDS-PAGE followed by Western blot staining using rabbit anti-human C3 (Santa Cruz) as a primary antibody and goat anti-rabbit-HRP conjugate (Dako) as a secondary antibody.

## Results

### In a solid phase binding assay, C1q binds to PLY in an antibody dependent fashion and promotes complement activation via the CP

**Figure 1:**
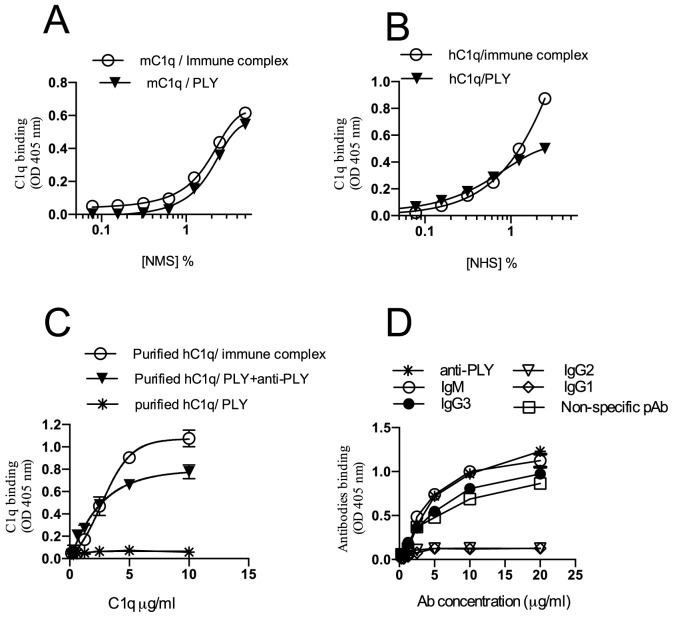
C1q binding to recombinant PLY exclusively occurs when either a PLY specific antibody or IgG3 or IgM binds to PLYin a non-specific fashion. Microtiter ELISA plates were coated either with PLY or immune complexes (positive control) formed by incubation of HSA and a polyclonal goat anti-HSA antibody to capture C1q from NMS (A) or NHS (B). Purified human C1q was serially diluted in BBS and incubated for 1 hr at RT in ELISA plates pre-coated with PLY. Purified C1q showed strong binding to PLY in the presence of a specific anti-PLY antibody, but not in the absence of immunoglobulins (C). Non-specific polyclonal antibodies of either all isotypes or isotype-specific preparations of IgM or IgG subclasses were serially diluted and incubated in PLY-coated ELISA plates. PLY binds non-immune IgM and IgG3, but does not bind IgG1 or IgG2 (D). Results are means (±SEM) of 3 independent experiments.

Binding of C1q to PLY was assessed in a solid phase binding ELISA. When exposing NHS or WT mouse sera (NMS) to solid phase bound recombinant PLY, C1q binding to PLY was even detected in high serum dilutions (see figs. 1a, b). The ability of PLY to capture C1q from serum was similar to that of immune complex coated plates used as a positive control. In order to assess whether C1q binds directly to PLY also in absence of immunoglobulins, serial dilutions of purified human C1q were incubated with PLY as well as with PLY that was pre-incubated with PLY specific monoclonal antibodies to allow the formation of immune complexes used here as a positive control. The results shown in figures 1c clearly demonstrate that on its own, C1q has no direct binding affinity to PLY. Following previous reports that described CP activation on PLY pre-incubated with polyclonal non-immune serum IgG preparations (Mitchell *et al.*, 1991), we aimed to identify which IgG subclass (es) mediate(s) this non-specific binding to PLY and whether or not the potent CP activating immunoglobulin class IgM may also bind to PLY in a non-specific, but high affinity manner. Figure 1c shows that purified human C1q does not bind to PLY in absence of immunoglobulins, C1q binding to PLY is therefore strictly dependent on the presence of either an anti-PLY monoclonal antibody or on immunoglobulins of pre-immune sera that non-specifically bind to PLY. Figure 1d demonstrates that amongst the non-immune immunoglobulins tested, only the IgG subclass IgG3 or IgM bind to PLY, while IgG1 and IgG2 antibodies (which can form immune complexes that bind C1q and activate the CP) show no specific binding to PLY. Since it is well established that IgG4 has no exposed interaction site for C1q binding (and this does not initiate CP activation), it was not included in this analysis. Although IgA is known not to activate the CP, its binding to PLY was also assessed revealing that IgA has no binding affinity to PLY (data not shown).

### Complement activation on PLY monitored by the detection of C3 cleavage products in solid phase and fluid phase assays

**Figure 2:**
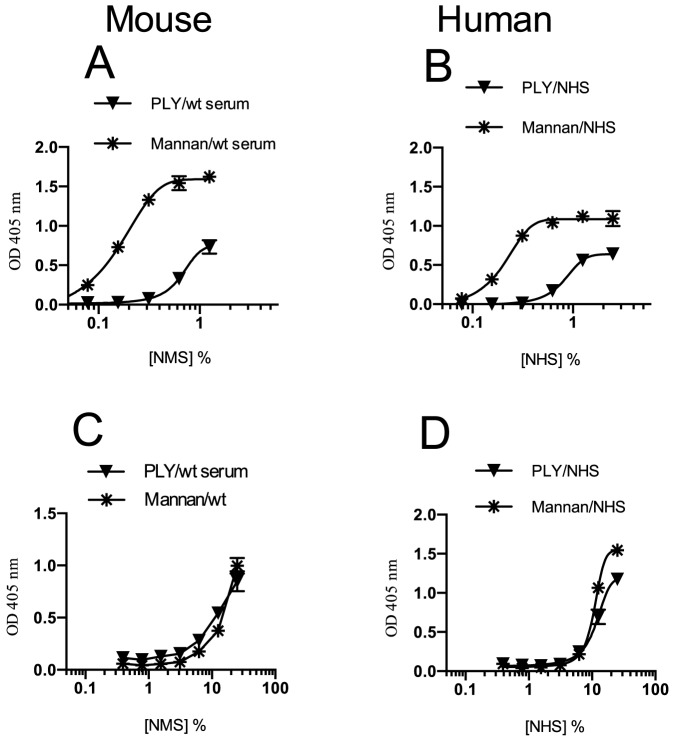
Deposition of C3 activation products, C3b and iC3b, on PLY. Serial dilutions of NMS or NHS in BBS were incubated in microtiter ELISA plates pre-coated with either PLY or mannan (positive control). ELISA plates were incubated for 1 hr at 37°C. The degree of C3b or iC3b deposition was monitored using a rabbit anti-human C3 antibody (as described in Materials and Methods). In both mouse (A) and human (B) sera, a significant degree of C3 activation occurred on PLY. This was seen even at high serum dilutions where complement activation can only occur by either the LP or the CP. At high serum concentration, using Mg^+2^-EGTA buffer (which inhibits C3 deposition via the calcium-dependent LP & CP), AP-mediated deposition of C3 activation products on PLY was also detected in both NMS (C) and NHS (D). Results are means (±SEM) of 3 independent experiments.

Serial dilutions of NMS and NHS were incubated in ELISA plates coated with either recombinant PLY or mannan (as positive control). Complement activation was measured in a C3 deposition assay as described. Figs 2a and 2b (NMS and NHS) show that complement is activated even at high serum dilutions, which indicates that either the CP or the LP drives C3 deposition on PLY, since the AP is inactive at serum concentrations below 5%. Interestingly, C3 deposition is also seen in lower serum dilutions in Mg^+2^ EGTA buffer (in which both the LP and the CP are inactive) indicating that the AP also contributes to C3 deposition on PLY in serum with serum concentrations higher than 5% and above (see Figs 2c and 2d).

**Figure 3:**
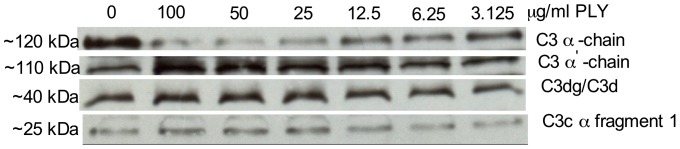
Western blot analysis to monitor C3 cleavage in response to the addition of increasing concentrations of PLY to NHS using a fluid phase assay. 1.25% NHS was incubated with serial dilutions of PLY in BBS buffer (starting concentration 100 µg/ml) for 1 hr at 37°C. Samples of this reaction were collected at different time points and subjected to a Western blot analysis using rabbit anti-human C3 to identify the various C3 activation products as described in Materials and Methods. A dose-dependent increase in the abundance of C3 activation products was observed to correlate with increasing PLY concentrations added to the serum.

To assess the ability of PLY to activate the complement cascade in the fluid phase (i.e. away from the bacterial surface) we incubated NHS with increasing concentrations of soluble PLY at 37°C. C3 degradation products were identified by Western blot analysis following SDS-PAGE under reducing conditions, filter blotting and staining of C3 fragments blotted to the filter membrane using a polyclonal anti-C3 antibody, as described in Materials and Methods. As shown in figure 3, the degree complement activation (monitored through the reduced intensity of the C3α chain band and increased abundance of C3 cleavage products) significantly increased with the concentration of PLY added to each serum sample. Our findings indicate that PLY can trigger C3 cleavage in the fluid phase, resulting in a significant degree of complement activation away from the pathogen surface.

**Figure 4:**
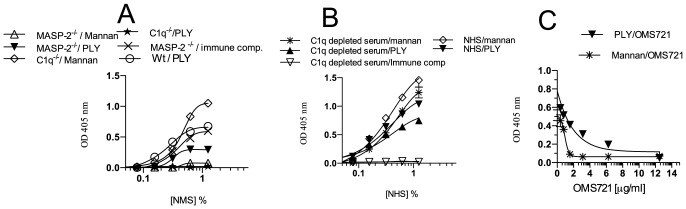
In C1q-depleted human serum, C3 can still be activated on PLY in a LP-dependent fashion. ELISA plates were either coated with PLY, or mannan, or immune complexes (as either positive or negative controls). (A) The deposition of C3 activation products on PLY was measured in serially diluted WT, C1q^−/−^ and MASP-2^−/−^ mouse sera. MASP-2 deficiency does not abolish C3 activation on PLY, while in the absence of C1q, no C3 activation can be detected in mouse sera. In human serum, however, absence of C1q has only a minor effect on the level of C3 deposition on PLY (**B**). In figure **C**, C1q-depleted human serum was incubated with different concentrations of anti-hMASP-2 mAb OMS721 followed by incubation with ELISA plates coated with PLY or mannan (as positive control) for 1 hr at 37°C. C3 deposition was detected using anti-human C3 as described in Materials and Methods. Inhibition of MASP-2 functional activity using the anti-hMASP-2 mAb OMS721 abolished C3 deposition on PLY in human C1q-depleted serum. Results are means (±SEM) of 3 independent experiments.

We further assessed sera from gene-targeted mouse lines with selective deficiencies for C1q (i.e. CP deficient) or MASP-2 (i.e. LP deficient) for possible defects in their ability to drive complement activation on PLY. C1q^−/−^ mouse serum failed to initiate complement activation and C3 deposition on PLY, while MASP-2^−/−^ mouse serum showed a reduced, but clearly detectable degree of C3 deposition on PLY-coated surfaces when compared to WT serum run in the same assay (see fig 4a). Our finding implies that in the mouse, the LP is not the major mechanism that drives complement activation on PLY.

In human serum, however, there is a completely different scenario: As shown in figure 4b, C1q-depleted human serum fails to deposit C3 on immune complexes, but shows no marked defect in its ability to deposit C3 on PLY. Since the serum dilutions chosen are too high for the AP to contribute to C3 deposition in C1q-depleted serum (which is CP deficient), the only activation pathway that could account for the high residual degree of C3 deposition at the given serum dilutions is the LP. In order to test this assumption, we added increasing concentrations of our recombinant antibody-based LP inhibitor OMS721. This mAb specifically binds MASP-2, the essential effector enzyme of the LP, and depletes LP functional activity [17]. As shown in figure 4c, MASP-2 depletion effectively blocks C3 deposition in C1q depleted human serum on mannan as well as on PLY-coated plates in an antibody concentration dependent manner. This underlines that the significant degree of C3 deposition on PLY seen in C1q-depleted NHS is mediated by the LP.

### Binding of LP carbohydrate recognition molecules to recombinant PLY

As a next step, we aimed to identify which of the LP-specific recognition subcomponents may directly bind to PLY to trigger C3 activation in a CP-independent fashion.

**Figure 5:**
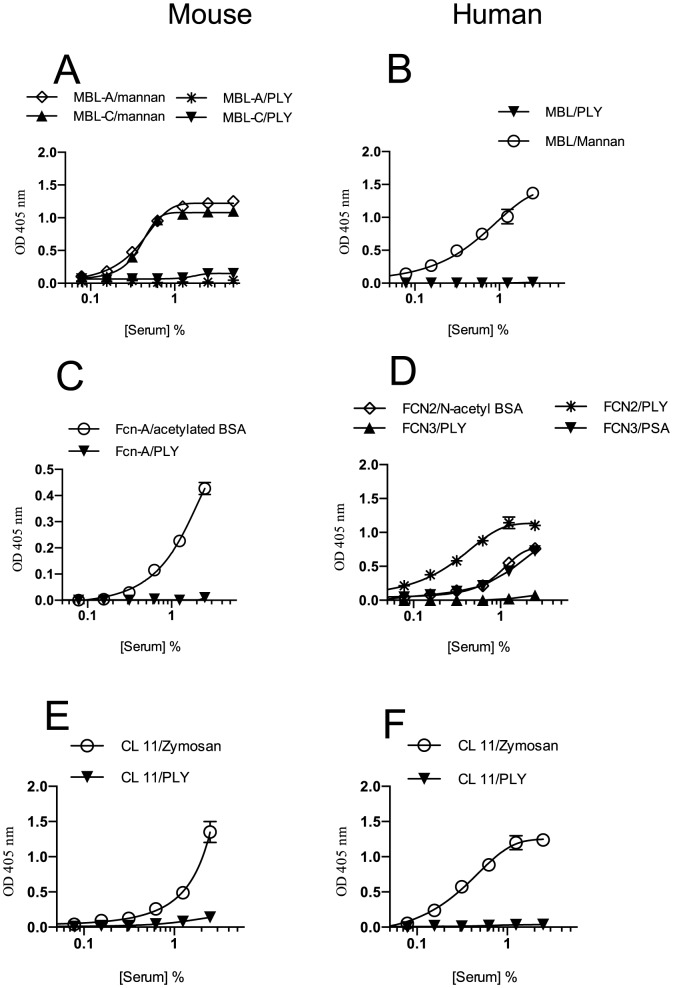
Human L-ficolin is the only LP recognition molecule that binds to PLY. Serial dilutions of NMS or NHS in BBS were incubated in ELISA plates pre-coated with PLY or specific ligands for LP recognition molecules. The binding of specific LP recognition molecules to PLY was subsequently detected using antibodies directed against MBL, ficolins and CL-11. The binding of murine MBL-A and MBL-C (A), murine ficolin-A (C), murine CL-11 (E), human MBL (B), human FCN2 & 3 (D) and human CL-11 (F) were assessed by ELISA. In contrast to mouse ficolin-A (which has no binding affinity to PLY) human L-ficolin (FCN2) (the human orthologoue of murine ficolin-A) showed strong binding to PLY. Results are means (±SEM) of three independent experiments.

In order to identify the human and murine LP recognition molecules that can directly bind to PLY and activate the LP, microtitre ELISA plates were coated with either recombinant PLY or known specific ligands for each of the LP recognition subcomponents tested. The results demonstrate that neither human MBL nor mouse MBL-A or MBL-C showed any detectable binding affinity towards recombinant PLY (fig. 5a–b). Likewise, neither human H-ficolin (alias FCN3) nor CL-11 (sourced from NHS) nor murine CL-11 (sourced from NMS) showed any binding affinity to PLY (fig. 5d–f). The only LP recognition component that showed strong and specific binding interaction with PLY was human L-ficolin (alias FCN2), while the murine orthologue of L-ficolin, ficolin-A failed to bind to PLY directly (fig. 5c,d). Addition of N-acetylated BSA (a ligand for L-ficolin) at increasing concentations to C1q-depleted human serum inhibited C3 activation on PLY with 10 microgram/mL of *N-acetylated BSA* showing complete inhibition (data not shown).

## Discussion

PLY is a potent virulence factor produced by all clinical isolates of *S. pneumoniae*. The toxin is a cholesterol-dependent cytolysin (CDC) that binds to membranes of eukaryotic cells where it oligomerises to form pores in the cell membrane with subsequent cell lysis [21]. The cytotoxic activity of PLY plays an essential role for optimal multiplication and dissemination of pneumococci into the lungs and blood [22].

PLY-mediated complement activation has been attributed to CP activation, with several studies suggesting that PLY acts to protect *S. pneumoniae* from complement attack and opsonophagocytosis by depletion of complement within the environment of the pneumococcus [13], [22], [23]. Our present work aimed to shed more light on molecular events leading to complement activation on PLY. We were able to show for the first time that the potent CP-activating immunoglobulin class IgM can bind non-specifically to PLY and refined the previous observation that PLY non-specifically binds to non-immune IgG to drive CP activation [2], [13], [24] by showing that of all of the 4 human IgG subclasses, only IgG3 Initiates CP activation on PLY [2], [13], [24]. The differences in the binding affinities between the IgG subclasses to PLY may be mainly due to the length of the hinge regions in each subclass rather than due to different glycosylation pattern (since the glycosylation pattern are identical in all IgG subclasses (IgG are glycosylated at aspargine residue 297 at the CH2 region of the IgG)). While, IgG1 and IgG2 have a relatively short hinge region (15 and 12 amino acid residues respectively), IgG3 has the longest hinge region corresponding to 62 amino acid residues [25]. The heavy glycosylation pattern at CH2 region and the relatively short hinge region of IgG1 (15 aa) and IgG2 (12 aa) may hinder its binding to PLY. In contrast, the extraordinary long hinge region of IgG3 (62 aa) keeps the Fab fragment away from the Fc region of IgG3. This may make it possible for the Fc region to bind directly to PLY.

We have shown that in human serum, the LP recognition molecule L-ficolin (alias FCN2) drives the LP on PLY and subsequently demonstrated that C3 deposition on PLY in C1q-depleted human serum was MASP-2 dependent, since inhibition of LP functional activity through application of our recombinant anti human MASP-2 mAb (OMS 721) abolished any C3 deposition on PLY in C1q deficient human serum in a dose dependent manner (see fig. 4c).

In mouse serum, PLY failed to activate complement via the LP, an observation that is underlined by the failure to detect any binding activity of PLY with any of the murine LP recognition subcomponents, including the murine L-ficolin orthologue ficolin-A (fig. 5a,c,e).

Interestingly, we also found that PLY binds to IgM non-specifically leading to subsequent activation of the CP.

It was suggested previously that complement activation on PLY in the fluid phase might be protective since it consumes complement activation away from the bacterial surface of *S.pneumoniae* [2], [8]. In order to determine to what extent the CP affects C3 deposition on PLY, we measured the levels of C3 deposition on PLY using C1q deficient mouse serum and C1q depleted human serum.

In human serum, we clearly show, for the first time, that PLY is able to trigger complement activation via the LP. When using C1q depleted human serum, C3 deposition was not abolished, as it was in C1q^−/−^ mouse serum, due to binding of the human LP recognition molecule L-ficolin to PLY.

The failure of ficolin-A, the murine orthologue to L-ficolin to bind to PLY is in line with previously reported observations indicating that murine ficolin-A has distinct binding specificities when compared with those of its human orthologue L-ficolin [26]. X-ray crystallography has revealed that human L-ficolin has 4 potential binding sites that can bind to acetylated carbohydrates (N-acetyl glucosamine, N-acetylneuraminic acid) and neutral carbohydrates, such as β (1→3)-D-glucan. L-ficolin was also found to bind lipoteichoic acid, C-reactive protein, fibrinogen, fibrin, DNA, elastin and corticosteroid [26]. On the other hand, mouse ficolin-A has a limited binding spectrum to some acetylated carbohydrates and to elastin [27], [28].

Our findings provide more detailed answers to the question raised by Paton *et al.* in 1984 wondering by which mechanisms human serum intitates complement deposition on PLY in the absence of specific antibodies. We show that in human serum, PLY initiates LP-mediated complement activation via the specific binding of L-ficolin to PLY. CP activation in human serum depends either on the presence of specific antibodies or can be driven by the non-specific binding of PLY to non-immune IgG3 and/or IgM.

Surprisingly, PLY does not induce LP activation in mouse serum since none of the murine LP recognition molecules bind to PLY, while both the classical and the alternative pathways can activate C3 on PLY in mouse serum.

Previous *in vivo* studies have shown that PLY is naturally released by *S. pneumoniae* at relatively high concentrations shortly after infection [29] and that PLY deficient *S.pneumoniae* strains are far less pathogenic then their PLY positive WT confounders [8]. The *in-vitro* data presented here show that PLY induces a significant degree of complement activation (as monitored by the degradation of complement C3 in the fluid phase), even when PLY was added at relatively low concentrations (such as 3.125 µg/ml) (see fig. 3). Our findings are in strong support of the hypothesis that the ability of PLY to activate and consume complement may increase the pathogenicity of *S.pneumoniae* since it may protect the pneumococcus from opsonophagocytosis through host immune cells by directing complement deposition and opsonisation away from the bacterial surface.

## References

[1] Miller E, Waight P, Efstratiou A, Brisson M, Johnson A, et al.. (2000) Epidemiology of invasive and other pneumococcal disease in children in england and wales 1996–1998. Acta Paediatr Suppl 89: 11–16. 10.1111/j.1651-2227.2000.tb00776.x 11194790

[2] Yuste J, Botto M, Paton JC, Holden DW, Brown JS (2005) Additive inhibition of complement deposition by pneumolysin and PspA facilitates streptococcus pneumoniae septicemia. J Immunol 175: 1813–1819. 16034123 10.4049/jimmunol.175.3.1813

[3] Kyaw MH, Christie P, Clarke SC, Mooney JD, Ahmed S, et al (2003) Invasive pneumococcal disease in scotland, 1999–2001: Use of record linkage to explore associations between patients and disease in relation to future vaccination policy. Clin Infect Dis 37: 1283–1291. 14583860 10.1086/379016

[4] Ali YM, Lynch NJ, Haleem KS, Fujita T, Endo Y, et al (2012) The lectin pathway of complement activation is a critical component of the innate immune response to pneumococcal infection. PLoS Pathog 8: e1002793. 22792067 10.1371/journal.ppat.1002793 PMC3390405

[5] Gadd KJ, Reid KB (1981) The binding of complement component C3 to antibody-antigen aggregates after activation of the alternative pathway in human serum. Biochem J 195: 471–480. 7316962 10.1042/bj1950471 PMC1162911

[6] Jarva H, Janulczyk R, Hellwage J, Zipfel PF, Bjorck L, et al (2002) Streptococcus pneumoniae evades complement attack and opsonophagocytosis by expressing the pspC locus-encoded hic protein that binds to short consensus repeats 8–11 of factor H. J Immunol 168: 1886–1894. 11823523 10.4049/jimmunol.168.4.1886

[7] Brown JS, Hussell T, Gilliland SM, Holden DW, Paton JC, et al (2002) The classical pathway is the dominant complement pathway required for innate immunity to streptococcus pneumoniae infection in mice. Proc Natl Acad Sci U S A 99: 16969–16974. 12477926 10.1073/pnas.012669199 PMC139253

[8] Quin LR, Moore QC 3rd, McDaniel LS (2007) Pneumolysin, PspA, and PspC contribute to pneumococcal evasion of early innate immune responses during bacteremia in mice. Infect Immun 75: 2067–2070. 17220305 10.1128/IAI.01727-06 PMC1865685

[9] Li J, Glover DT, Szalai AJ, Hollingshead SK, Briles DE (2007) PspA and PspC minimize immune adherence and transfer of pneumococci from erythrocytes to macrophages through their effects on complement activation. Infect Immun 75: 5877–5885. 17923519 10.1128/IAI.00839-07 PMC2168335

[10] Kalin M, Kanclerski K, Granstrom M, Mollby R (1987) Diagnosis of pneumococcal pneumonia by enzyme-linked immunosorbent assay of antibodies to pneumococcal hemolysin (pneumolysin). J Clin Microbiol 25: 226–229. 3818919 10.1128/jcm.25.2.226-229.1987 PMC265872

[11] Tilley SJ, Orlova EV, Gilbert RJ, Andrew PW, Saibil HR (2005) Structural basis of pore formation by the bacterial toxin pneumolysin. Cell 121: 247–256. 15851031 10.1016/j.cell.2005.02.033

[12] Kadioglu A, Andrew PW (2004) The innate immune response to pneumococcal lung infection: The untold story. Trends Immunol 25: 143–149. 15036042 10.1016/j.it.2003.12.006

[13] Mitchell TJ, Andrew PW, Saunders FK, Smith AN, Boulnois GJ (1991) Complement activation and antibody binding by pneumolysin via a region of the toxin homologous to a human acute-phase protein. Mol Microbiol 5: 1883–1888. 1766369 10.1111/j.1365-2958.1991.tb00812.x

[14] Wallis R, Mitchell DA, Schmid R, Schwaeble WJ, Keeble AH (2010) Paths reunited: Initiation of the classical and lectin pathways of complement activation. Immunobiology 215: 1–11. 19783065 10.1016/j.imbio.2009.08.006 PMC2824237

[15] Venkatraman Girija U, Gingras AR, Marshall JE, Panchal R, Sheikh MA, et al (2013) Structural basis of the C1q/C1s interaction and its central role in assembly of the C1 complex of complement activation. Proc Natl Acad Sci U S A 110: 13916–13920. 23922389 10.1073/pnas.1311113110 PMC3752233

[16] Tsujimura M, Ishida C, Sagara Y, Miyazaki T, Murakami K, et al (2001) Detection of serum thermolabile beta-2 macroglycoprotein (hakata antigen) by enzyme-linked immunosorbent assay using polysaccharide produced by aerococcus viridans. Clin Diagn Lab Immunol 8: 454–459. 11238239 10.1128/CDLI.8.2.454-459.2001 PMC96080

[17] Schwaeble WJ, Lynch NJ, Clark JE, Marber M, Samani NJ, et al (2011) Targeting of mannan-binding lectin-associated serine protease-2 confers protection from myocardial and gastrointestinal ischemia/reperfusion injury. Proc Natl Acad Sci U S A 108: 7523–7528. 21502512 10.1073/pnas.1101748108 PMC3088599

[18] Tan LA, Yu B, Sim FC, Kishore U, Sim RB (2010) Complement activation by phospholipids: The interplay of factor H and C1q. Protein Cell 1: 1033–1049. 21153520 10.1007/s13238-010-0125-8 PMC4875149

[19] El Aswad Bel D, Doenhoff MJ, El Hadidi AS, Schwaeble WJ, Lynch NJ (2011) Use of recombinant calreticulin and cercarial transformation fluid (CTF) in the serodiagnosis of schistosoma mansoni. Immunobiology 216: 379–385. 20691496 10.1016/j.imbio.2010.06.014

[20] Lynch NJ, Roscher S, Hartung T, Morath S, Matsushita M, et al (2004) L-ficolin specifically binds to lipoteichoic acid, a cell wall constituent of gram-positive bacteria, and activates the lectin pathway of complement. J Immunol 172: 1198–1202. 14707097 10.4049/jimmunol.172.2.1198

[21] Gilbert RJ (2010) Cholesterol-dependent cytolysins. Adv Exp Med Biol 677: 56–66. 20687480 10.1007/978-1-4419-6327-7_5

[22] Berry AM, Ogunniyi AD, Miller DC, Paton JC (1999) Comparative virulence of streptococcus pneumoniae strains with insertion-duplication, point, and deletion mutations in the pneumolysin gene. Infect Immun 67: 981–985. 9916120 10.1128/iai.67.2.981-985.1999 PMC96416

[23] Rossjohn J, Gilbert RJ, Crane D, Morgan PJ, Mitchell TJ, et al (1998) The molecular mechanism of pneumolysin, a virulence factor from streptococcus pneumoniae. J Mol Biol 284: 449–461. 9813129 10.1006/jmbi.1998.2167

[24] Paton JC, Rowan-Kelly B, Ferrante A (1984) Activation of human complement by the pneumococcal toxin pneumolysin. Infect Immun 43: 1085–1087. 6698602 10.1128/iai.43.3.1085-1087.1984 PMC264298

[25] Phillips ML, Tao MH, Morrison SL, Schumaker VN (1994) Human/mouse chimeric monoclonal antibodies with human IgG1, IgG2, IgG3 and IgG4 constant domains: Electron microscopic and hydrodynamic characterization. Mol Immunol 31: 1201–1210. 7935507 10.1016/0161-5890(94)90034-5

[26] Matsushita M (2010) Ficolins: Complement-activating lectins involved in innate immunity. J Innate Immun 2: 24–32. 20375620 10.1159/000228160

[27] Ohashi T, Erickson HP (1998) Oligomeric structure and tissue distribution of ficolins from mouse, pig and human. Arch Biochem Biophys 360: 223–232. 9851834 10.1006/abbi.1998.0957

[28] Fujimori Y, Harumiya S, Fukumoto Y, Miura Y, Yagasaki K, et al (1998) Molecular cloning and characterization of mouse ficolin-A. Biochem Biophys Res Commun 244: 796–800. 9535745 10.1006/bbrc.1998.8344

[29] Feldman C, Mitchell TJ, Andrew PW, Boulnois GJ, Read RC, et al (1990) The effect of streptococcus pneumoniae pneumolysin on human respiratory epithelium in vitro. Microb Pathog 9: 275–284. 2097494 10.1016/0882-4010(90)90016-j

